# Mirror-normal difference in the late phase of mental rotation: An ERP study

**DOI:** 10.1371/journal.pone.0184963

**Published:** 2017-09-15

**Authors:** Cheng Quan, Chunyong Li, Jiguo Xue, Jingwei Yue, Chenggang Zhang

**Affiliations:** Beijing Institute of Radiation Medicine, State Key Laboratory of Proteomics, Cognitive and Mental Health Research Center, Beijing, P.R., China; The University of Auckland, NEW ZEALAND

## Abstract

Mirror-normal letter discriminations are thought to require mental rotation in order to transform the rotated alphanumeric character into its canonical orientation. Moreover, out-of-plane rotation is likely to occur after in-plane rotation to fully normalize the mirror version before the final mirror-normal judgment. The so-called rotation-related negativity, which varies with orientation, is found in both ERP_onset_ (averaged with respect to stimulus onset) and ERP_RT_ (averaged with respect to response time), representing the involvement of mental rotation in both time windows. Additionally, the mean amplitude of ERP_RT_ correlates with individual performance. We performed a comprehensive analysis of the mirror-normal differences in the early and late phases of mental rotation and deduced that out-of-plane rotation is more likely to occur in the late phase and interacts with both in-plane rotation and the decision-making process, as indicated by both behavioral and electrophysiological findings.

## Introduction

Mental rotation is the ability to imagine the representation of a two- or three-dimensional object turning in the mind [1]. The purpose of the study of mental rotation is to evaluate individual visuo-spatial ability and to explore the process of visual imagery and spatial cognition [2–4]. One of the most commonly used paradigms for testing mental rotation was proposed by Cooper and Shepard in 1973, in which subjects are instructed to determine whether an alphanumeric character that is tilted away from its canonical orientation, is presented as its normal or mirror version [5]. Due to the existence of the well-learned canonical orientation of characters that involves both the vertical and the horizontal directions, this paradigm shows only one character during a single trial. Additionally, a shorter response time (RT) is required relative to other types of stimuli, such as three-dimensional block figures or polygons [1, 6, 7]. The response time increases monotonically with the angular displacement from the canonical orientation, which has been taken as evidence that subjects make the final mirror-normal judgment after mentally rotating the stimuli into the canonical orientation [5, 8–10]. In contrast to three-dimensional block figures, the RT function in the mirror-normal letter discrimination task departs from linearity and contains another quadratic component [9, 11]. It has been suggested that this phenomenon results from the familiarity of the stimuli. When the stimulus is familiar, it tends to be overlearned and achieves a certain degree of indifference to small angular deviations from its canonical orientation [5, 12]. In other words, the mean RT function represents a mixture of both rotation and non-rotation trials [13]. Kung and Hamm suggest that less spatial conflict between the polarity of the character-centered and viewer-centered coordinate system in non-rotation trials makes it possible for subjects to respond without mental rotation by simply comparing the vertical and horizontal polarity of the character [13, 14].

Event-related potentials (ERP) with high time resolution are an extremely effective tool to investigate the neural mechanism underlying mental rotation [15]. Mental rotation is associated with the amplitude modulation of ERPs, called the rotation-related negativity. The negativity usually appears at 400 ms post-stimulus over the parietal scalp and varies as a function of the rotation angle, which becomes a specific correlate of the mental rotation process [11, 16–21]. However, the slow wave remains positive because it is superimposed on the simultaneously prevailing P300 [19, 22]. Interestingly, when Riečanský replicated these characteristics in the early phase (ERP_onset_, time-locked to stimulus onset) and late phase (ERP_RT_, time-locked to response time) of mental rotation, he found that ERP_RT_ is not only associated with the orientation but is also related to the response time [2]. This finding suggests that the processes of mental rotation and decision-making coexist in the late phase [2]. Considering that the RT function in the mirror-normal letter discrimination task could be described by the Mixture Model of three parameters, including the baseline RT, the rotation rate and the proportion of trials employing mental rotation [13, 14, 23], the involvement of decision-making process makes the late phase of mental rotation important for examining the mixture of rotation and non-rotation trials. The absence of a relationship between ERP_onset_ and response time offers an alternative interpretation of the amplitude modulation in ERP_onset_: the orientation effect in ERP_onset_, which indicates more negative ERP detection and smaller absolute amplitude with larger orientation, could result from the increased duration/latency jitter among single trials [19, 24, 25]. A true correlate of the rotation process would also cause the amplitude to vary with individual performance [2].

In addition to the orientation effect, the mirror-normal difference is another important issue in mental rotation [26]. It is critical to investigate how different versions of stimuli (mirror or normal version) affect the process of mental rotation [8, 9, 26–28]. In a traditional test of mental rotation, the response time to the mirror version of stimuli is usually longer than that to the normal version. To interpret the extra response time in the mirror trials, Cooper and Shepard first proposed a hypothesis that the subjects’ tendency to prepare a normal response prolongs the response time to the mirror version [1]. However, the ERP difference between upright normal and upright mirror trials was confirmed to be similar to the ERP difference between upright normal and inverted normal trials, which suggests that upright mirror letters are being rotated [9]. As a result, assumptions based on ‘flip’ gradually emerged and provide another explanation for the mirror-normal difference in the discrimination task. Flipping involves the process of rotation out of the plane after 2D rotation in the plane to fully normalize the mirror version, which prolongs the response time [9]. Furthermore, the discovery of the relationship between the rotation rates and the response time of mirror-normal differences confirms the hypothesis of flipping [9, 29]. The results of positron emission tomography (PET) also confirmed that the activation of brain areas in the upright mirror version was consistent with the activation areas during the traditional test of mental rotation [30]. It is generally considered that flipping occurs after in-plane rotation, given the contradiction that if out-of-plane rotation occurs before in-plane rotation, there is no need to apply 2D rotation because the characteristics of different versions have already been dissociated in the mind [9, 11, 28]. Further analysis showed that the ERP differences between mirror and normal trials are delayed as the orientation increases, supporting the assumption that out-of-plane rotation occurs after in-plane rotation [9, 28]. However, for large orientations, rotation in and out of the plane probably occurs in parallel and obscures the mirror-normal difference [28]. Moreover, it is difficult to validate the existence of flipping in paradigms with three-dimensional objects, suggesting that out-of-plane rotation requires a holistic mental rotation process rather than an analytic process [28, 31]. Different processes might also lead to controversy concerning whether mental rotation has a left or right hemisphere specialization [18, 26, 32–35]. Furthermore, flipping does not occur in the left/right facing task which is quite similar to the mirror-normal letter discrimination task except that different horizontal direction (left or right facing) does not matter with typically common objects [23]. Therefore, out-of-plane rotation might be unique to the mirror-normal letter discrimination task because alphanumeric characters have a well-learned canonical representation that involves both the up/down and the left/right dimension.

Most studies investigating the mirror-normal difference of mental rotation have focused on ERP_onset_ and neglected the observation that the orientation effect also appears in ERP_RT_ [2]. Moreover, there is a much more significant association with individual performance in the late phase of mental rotation. If out-of-plane rotation does exist and tends to shift earlier for less rotated stimuli [9], this flip component in mirror trials would line up with various processes in normal trials for different orientations, which could complicate the analysis of the mirror-normal difference through ERP_onset_. In contrast, the orientation effect on the post-rotation process is clearly less than that on the rotation process [2, 16, 36], which suggests that the flip component in mirror trials would line up with just the in-plane rotation in normal trials for each orientation if the mirror-normal differences were examined through ERP_RT_. As a result, studying the mirror-normal difference in ERP_RT_ has the advantage of making a more direct comparison of the in-plane and out-of-plane rotation than ERP_onset_ and avoids the problems caused by the fact that post-rotation processing begins at different time points for different orientations. Above all, a detailed analysis of the mirror-normal difference in ERP_RT_ will precisely reveal the differences between the in-plane and out-of-plane rotation and contribute to a comprehensive understanding of flipping in the mirror version of stimuli and the relationship between ERP and individual performance. Because many previous studies on ERP_onset_ have indicated that flipping should occur in the late phase of mental rotation, the ultimate goal of our study was to confirm this hypothesis by comparing the orientation effect and the mirror-normal difference between the early and late phases of the mirror-normal letter discrimination task, which could contribute to the theoretical proof of out-of-plane rotation from a unique perspective. Based on previous studies, consistent brain activation during mental rotation is mainly distributed over parietal regions [10, 22, 33, 37, 38]. Thus, we focused on the parietal region and replicated previous methods used for ERP_onset_ research. First, we thoroughly explored the differences in RT functions and ERP patterns between the mirror and normal versions for both ERP_onset_ and ERP_RT_ and then performed a detailed analysis of every 50-ms time window to further test whether there was a delay in the mirror-normal differences in the late phase of mental rotation.

## Methods

### Subjects

Twenty-seven healthy volunteers (26 males, 1 female, all right-handed) participated in the study; their ages ranged from 22 to 42 (27.81±3.72). They were all postgraduates and had normal or corrected-to-normal vision. To reduce the potential interaction of the orientation and the horizontal direction (the characters R, F, and 5 face to the right in normal trials and left in mirror trials, whereas the characters 2 and 4 face to the left in normal trials and right in mirror trials), subjects were required to meet the criteria for a mean accuracy of at least 85% and mean accuracies of at least 65% for both mirror or normal trials and right-facing or left-facing trials for each orientation [14]. Data from five subjects were excluded from the analysis due to difficulty in removing artifacts from recordings or failure to meet accuracy requirements. Thus, twenty-two subjects (22 males) were included in the final analysis, ranging in age from 22 to 42 (28.09±3.96). This study was approved by the Ethics Committee of Beijing Institute of Radiation Medicine, and all volunteers provided written informed consent to participate in this experiment.

### Visual stimuli

**Fig 1:**
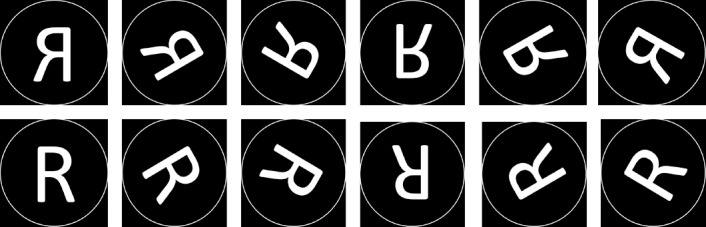
Mirror and normal versions of the letter R at each orientation (from 0° to 300°).

Two uppercase letters (F, R) and three digits (2, 4, 5) were printed in white 72-point Arial font and presented on a black background in their normal and mirror versions at four orientations (0°, ±60°, ±120°, and 180°) (see Fig 1). In the upright orientation, the characters subtended a vertical visual angle of 6.1° and a horizontal visual angle of 4.1°. Stimuli were displayed on an SVGA monitor (1920 x 1080 pixel resolution; 60-Hz refresh rate) from a distance of 65 cm and manipulated by the PSYCHTOOLBOX in MatLab. Transistor-transistor logic (TTL) pulses generated via the serial port of the display computer provided synchronization of stimulus events with electroencephalogram (EEG) acquisition.

### Procedure

Before the regular trials, participants practiced the mirror-normal letter discrimination task with two training blocks of 40 trials. The practice ended as soon as the participant had met the minimum accuracy of 90% in each training block. In the regular experiment, each participant was given five blocks of 80 trials. The sequence of stimulus conditions was randomized within the blocks. Each block included a combination of factors as follows: orientation (0°, ±60°, ±120°, or 180°), character version (normal or mirror version) and type of stimulus (five alphanumeric characters). As in the previous study, rotations of the stimulus in clockwise and counter-clockwise directions were considered equivalent and there were twice as many stimuli at an orientation of 0° or 180° as there were stimuli at an orientation of 60° or 120°, either in a clockwise or counter-clockwise direction. All subjects were tested in 400 trials, 50 for each experimental condition, resulting from the combination of orientations (0°, 60°, 120°, or 180°) and character versions (normal or mirror version).

Each trial began with a white fixation plus sign presented at the center of the black background for 1000 ms. Then, a character appeared on the screen and remained for 4 s or until the subject reacted. Subjects were instructed to make a mirror-normal discrimination of the character and to respond by pressing 1 (stands for normal version) or 2 (stands for mirror version) on the numeric keypad of the computer keyboard as quickly and as accurately as possible. Subjects were instructed to respond with two fingers of their right hands to achieve a quick response and most subjects used the index and middle finger for convenience. Subjects were also encouraged to avoid eye movements during the recording period and to make any eye-blinks during the pauses between trials.

### EEG apparatus and pre-processing

**Fig 2:**
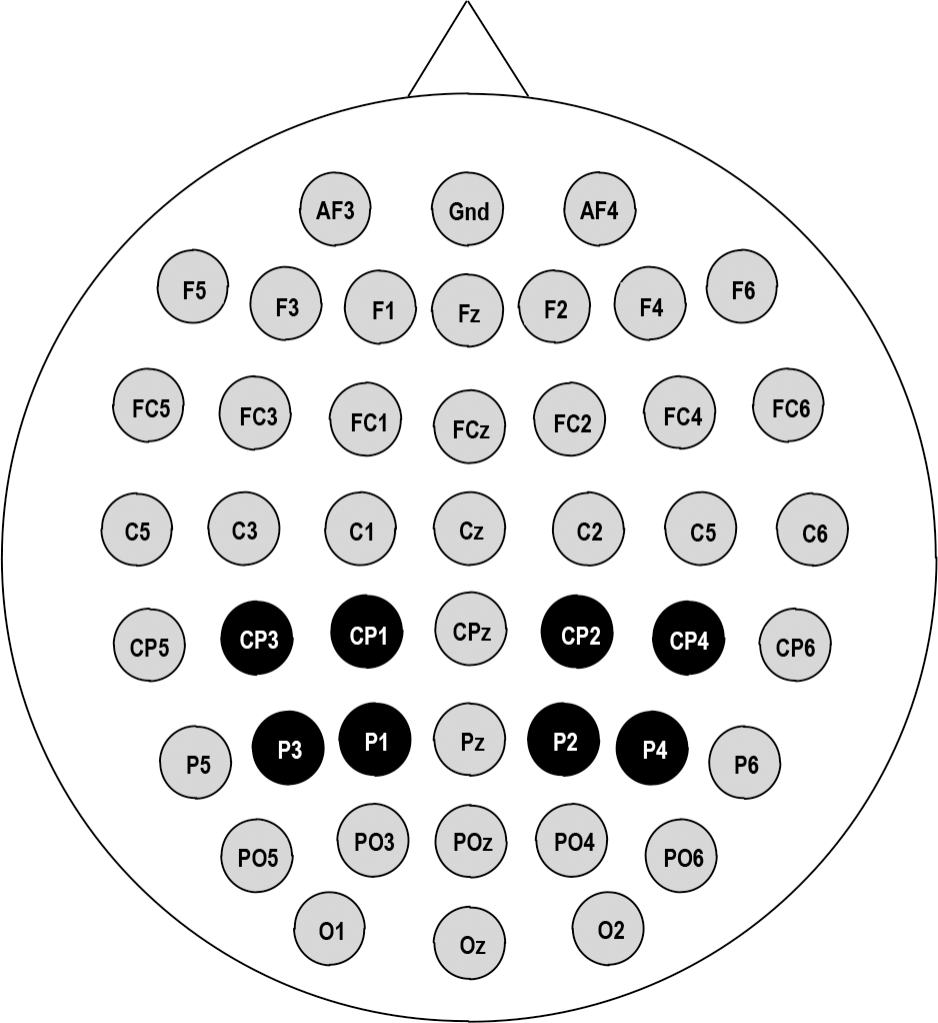
Forty-five EEG electrode locations. The eight parietal electrodes in shades of black were used in the ERP amplitude analysis. All electrodes were included in the topographic mappings.

A 128-channel neural signal processor (Blackrock microsystems LLC) was used. Sixty-four electrodes were positioned according to the 10–20 International System. The EEG of 45 electrodes (see Fig 2) was recorded continuously (1000 Hz sampling rate, 0.1–500 Hz analogue band-pass) with a neural signal amplifier (Blackrock microsystems LLC) using a common vertex (Cz) reference, and re-referenced to the average reference off-line. For all electrodes, impedances were maintained below 5K ohms.

Only the trials with correct responses and a recording length between 600 and 4000 ms were included in the analysis. The EEG files were digitally filtered in the range of 1–45 Hz and corrected for eye movement by artifact subspace reconstruction in the EEGLAB toolbox of MatLab [39, 40]. In a previous study, trials with a response time greater than 1500 ms had to be removed from ERP_RT_ when the data were fixed with respect to the event target [2, 41]. To avoid invalidating too many trials, we instead divided the calculation of ERP into two steps, and then all trials with a response time lower than 4000 ms could be reserved. First, the corrected data were segmented with respect to the event target over 2000 ms, spanning from -500 to 1500 ms, and the DC offset was calculated from the pre-stimulus baseline (-500 to 0 ms). The segmented data were then averaged to produce a total of 8 ERP_onset_s (two character versions and four orientations) relative to the DC offset. Secondly, the original corrected data (before segmentation) were segmented with respect to the event response over 2000 ms, spanning from -1500 to 500 ms and subtracting the same DC offset of ERP_onset_ for each trial. Segmented data in step two were then averaged to produce another eight ERP_RT_s for each subject. The numbers of trials ended up being averaged for each condition are listed as follows: 39±9(0°), 43±8(60°), 47±2(120°), 44±2(180°) in the normal version; 47±4(0°), 48±1(60°), 47±2(120°), 42±4(180°) in the mirror version. In addition, the mean amplitude of the intervals 400–600 ms from stimulus onset and 600–200 ms before the response were computed for further statistical analysis.

### Data analysis

Two 2 x 4 repeated-measures analyses of variance (ANOVA) were calculated for response time (including trials with a correct response) and accuracy (including all trials) using the character version (normal and mirror) and orientation (0°, 60°, 120°, and 180°) as within-subject factors. The Greenhouse-Geisser correction for sphericity departures was applied when appropriate. Pairwise tests were performed after a significant main effect was confirmed. Simple effect tests were conducted in the presence of a significant interaction. Orthogonal polynomial contrasts were used to discover the linear or quadratic trend of variables. The rotation rates were estimated as the slope of the regression line for the response time over the orientation. Correlations between rotation rates and the magnitude of the mirror-normal difference were assessed by Pearson’s correlation coefficient.

The EEG analysis process was similar to that described in experiments conducted in 2009 [28]. Eight electrodes in the parietal region (CP3, CP1, CP2, CP4, P3, P1, P2, and P4) were considered for statistical analysis. Another 2 x 2 x 4 repeated-measures ANOVA was calculated for the mean ERP amplitude of intervals 400–600 ms from the stimulus onset and 600–200 ms before the response using laterality (left and right), character version (normal and mirror) and orientation (0°, 60°, 120°, and 180°) as within-subject factors. The mean ERP amplitude was averaged over electrodes of each hemisphere (Left hemisphere: CP3, CP1, P3, P1; Right hemisphere: CP2, CP4, P2, P4). Methods of statistical analysis were consistent with methods for behavioral data. Additional analysis of the rotation coefficient between the mean ERP amplitude and individual performance was assessed by Pearson’s correlation coefficient. For each correlation analyses for response time or ERP amplitude, the possible influential data pairs were identified by calculating Cook’s Distance based on linear regression. We then removed outliers that showed a Cook’s Distance greater than 4/N, where N is the number of observations, and recalculate the correlation coefficient based on the remaining pairs [42]. A more detailed analysis of the mirror-normal difference was performed to detect whether delays in the mirror-normal difference across orientations could appear at the interval of 600–200 ms before the response. Repeated-measures ANOVAs were performed for the mean ERP amplitude in 50-ms time windows in this interval for each orientation, taking character version (normal and mirror) and laterality (left and right) as within-subject factors.

## Results

### Behavioral data

**Fig 3:**
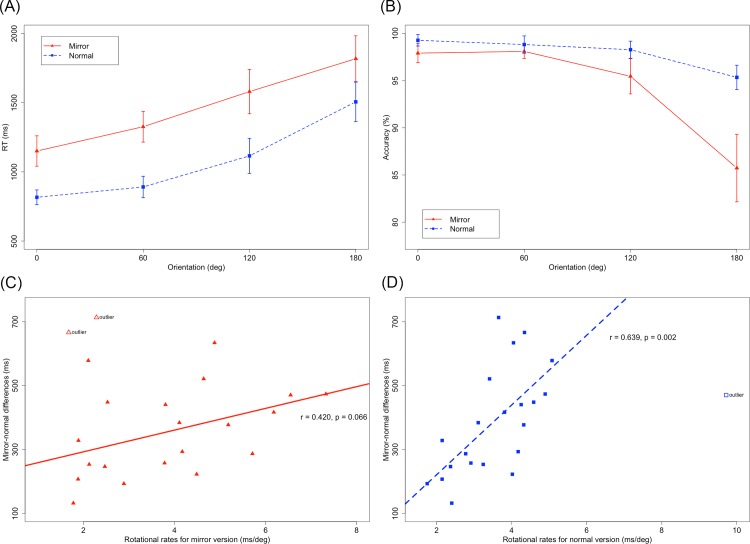
(A) The mean response time as a function of version and orientation. (B) The accuracy as a function of version and orientation. (C) Association of the individual mean mirror-normal difference in response time and rotation rates in the mirror version. (D) Association of the individual mean mirror-normal difference in response time and rotation rates in the normal version.

For response time, the main effects of character version (F(1,21) = 121.841, p < 0.001, η^2^ = 0.308, MSE = 0.054) and orientation (F(3,63) = 105.532, p < 0.001, ε = 0.559, η^2^ = 0.444, MSE = 0.037) were significant, as was the interaction between character version and orientation (F(3,63) = 4.715, p = 0.018, ε = 0.583, η^2^ = 0.012, MSE = 0.013). Responses to normal characters (0°: 816±127 ms, 60°: 891±185 ms, 120°: 1115±303 ms, 180°: 1506±341 ms) were faster than those to mirror characters (0°: 1151±263 ms, 60°: 1326±266 ms, 120°: 1580±383 ms, 180°: 1817±396 ms) (p < 0.001). The orientation effect could be described by a linear and quadratic trend in the normal version (F(1,63) = 265.930, p < 0.001 for the linear trend, F(1,63) = 25.223, p < 0.001 for the quadratic trend, accounting for 91.3% and 8.6% of the variance) and a linear trend in the mirror version (F(1,63) = 196.270, p < 0.001, accounting for 99.4% of the variance). The difference between any two orientations was significant for both the mirror and the normal data separately (all p < 0.01, see Fig 3(A)), excluding the combination of 0° and 60° in the normal version (p > 0.05).

In terms of accuracy, the main effects of character version (F(1,21) = 26.079, p < 0.001, η^2^ = 0.183, MSE = 0.002) and orientation (F(3,63) = 53.573, p < 0.001, ε = 0.646, η^2^ = 0.423, MSE = 0.001) were also significant, as was the interaction between version and orientation (F(3,63) = 15.950, p < 0.001, ε = 0.549, η^2^ = 0.175, MSE = 0.001). Overall, the accuracy was better in the normal (0°: 99.2%±1.4%, 60°: 98.8%±2.1%, 120°: 98.2%±2.1%, 180°: 95.3%±3.0%) than in the mirror version (0°: 97.9%±2.4%, 60°: 98.0%±1.7%, 120°: 95.4%±4.4%, 180°: 85.7%±8.5%) (p < 0.001). The orientation effect could be described by a linear and quadratic trend in both the normal (F(1,63) = 35.052, p < 0.001 for linear trend, F(1,63) = 7.057, p = 0.010 for quadratic trend, accounted for 80.9% and 16.3% of variance) and mirror (F(1,63) = 90.910, p < 0.001 for linear trend, F(1,63) = 29.060, p < 0.001 for quadratic trend, accounted for 75.1% and 24.0% of variance) versions. Tests of simple effects found that the accuracy of inverted orientations was worse than that of any other orientation for both versions (all p < 0.001). Differences between other orientations did not approach significance for each version (all p > 0.05, see Fig 3(B)).

One factor that supports out-of-plane rotation is the relationship between rotation rates and the magnitude of the mirror-normal differences in response time. However, there was no correlation when the rotation rates were estimated as the slope of the linear component of the response time function across the mirror and normal versions (p > 0.05). A correlation could be found as long as the rotation rates were estimated in the normal version (r = 0.461, p = 0.031 before outlier detection; r = 0.640, p = 0.002 after outlier detection) alone instead of as mixed versions (p > 0.05) or merely the mirror version (p > 0.05) (see Fig 3(C) and 3(D)). In addition, neither the mirror-normal difference nor the individual rotation rate in the normal version was correlated with response time for the upright normal version (all p > 0.05), suggesting that this correlation in the normal version was not related to personal general processing efficiency [9, 13]. Moreover, the mean response time was negatively correlated with the individual accuracy (r = -0.465, p = 0.029), suggesting that the results were not due to the speed/accuracy trade-off [14]. The correlation remained significant in the mirror (r = -0.496, p = 0.022) and normal (r = -0.451, p = 0.035) versions.

### EEG results

**Fig 4:**
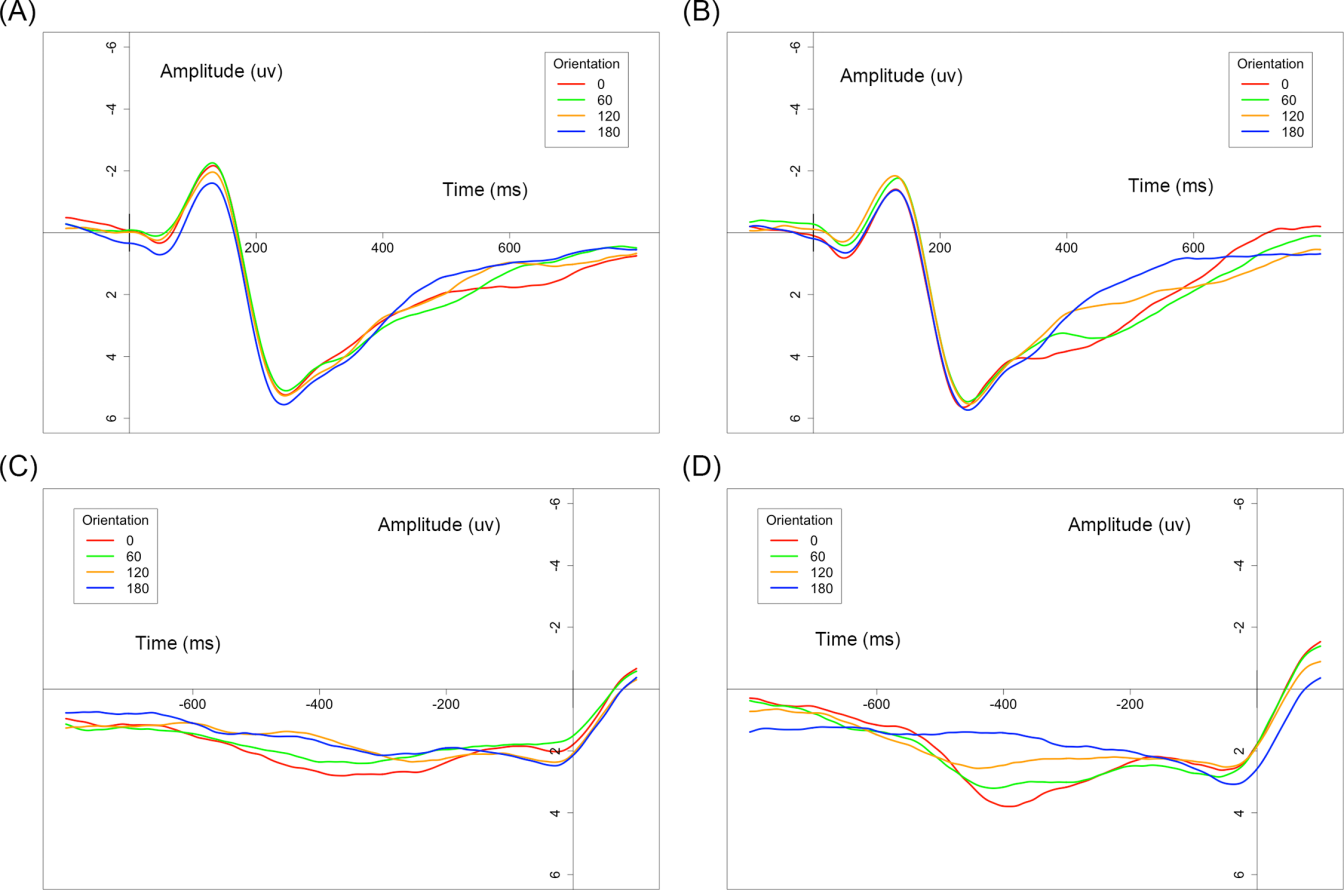
Average ERPs for the parietal region with respect to (A) stimulus onset in the mirror version, (B) stimulus onset in the normal version, (C) response time in the mirror version, and (D) response time in the normal version.

**Fig 5:**
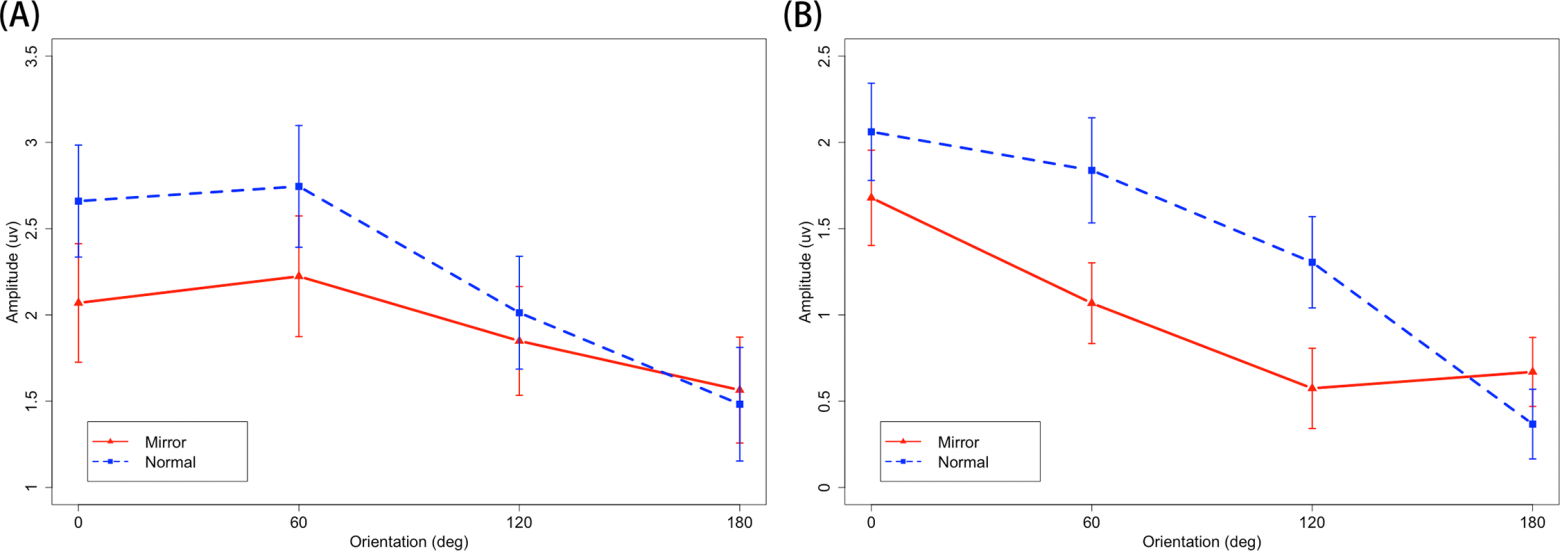
Mean ERP amplitude as a function of version and orientation over the parietal region in the interval (A) 400–600 ms from stimulus onset and (B) 600–200 ms before the response.

Fig 4 shows the average ERPs with respect to stimulus onset and response time for each orientation of the normal and mirror versions. Fig 5 shows the mean ERP_onset_ and ERP_RT_ amplitude for the mirror and normal versions as a function of orientation. Only the effects that were relevant to the interests for our study are reported.

**Fig 6:**
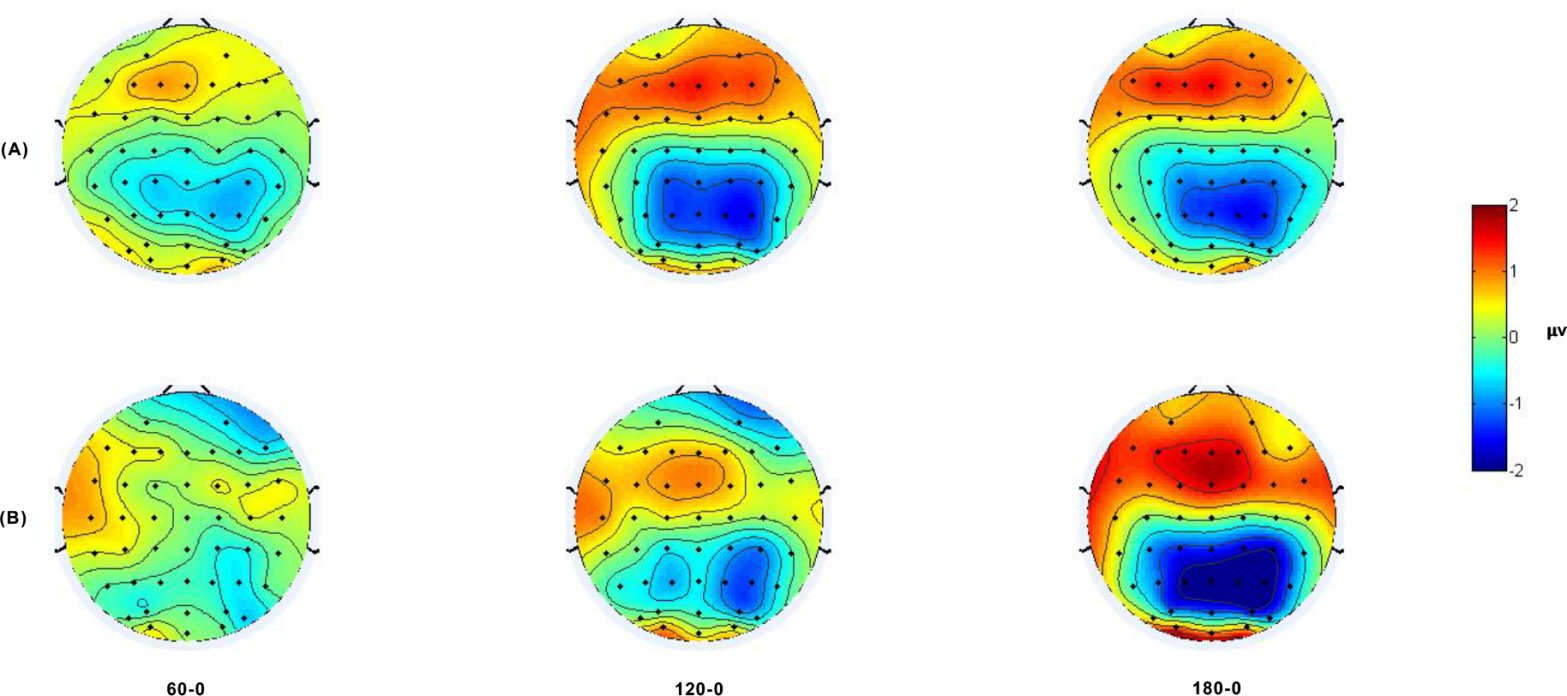
**Topographic mapping of the mean differences in amplitude in the interval of 600–200 ms before the response (A) in the mirror version and (B) in the normal version.** From left to right, voltage differences between 60°, 120°, 180° and the 0°.

A summary of the significant effects is presented in Table 1. ANOVA analysis showed the main effect of character version and orientation for both ERP_onset_ and ERP_RT_. Both the mean amplitude of 400–600 ms of ERP_onset_ (0°: 2.659±2.197 μV, 60°: 2.744±2.389 μV, 120°: 2.012±2.211 μV, 180°: 1.482±2.229 μV in the normal version; 0°: 2.069±2.322 μV, 60°: 2.223±2.366 μV, 120°: 1.849±2.133 μV, 180°: 1.564±2.079 μV in the mirror version) and 600–200 ms of ERP_RT_ (0°: 2.061±1.905 μV, 60°: 1.837±2.061 μV, 120°: 1.305±1.788 μV, 180°: 0.367±1.367 μV in the normal version; 0°: 1.678±1.869 μV, 60°: 1.068±1.583 μV, 120°: 0.574±1.574 μV, 180°: 0.669±1.353 μV in the mirror version) in the normal version were larger than those in the mirror version (p = 0.031 for ERP_onset_, p < 0.001 for ERP_RT_). A significant interaction of character version and orientation was found only for ERP_RT_. For ERP_onset_, differences between the orientations of 0°-180° (p < 0.001), 60°-120° (p = 0.021) and 60°-180° (p < 0.001) were found, and the orientation effect could be described by a single linear trend. Differences between other orientations did not approach significance (all p > 0.05). In terms of ERP_RT_, the simple effects test of interaction of character version and orientation showed that the orientation effect was significant for both the mirror (F(3,63) = 11.027, p < 0.001, ε = 0.740, η^2^ = 0.085, MSE = 1.005) and normal versions (F(3,63) = 28.298, p <0.001, ε = 0.704, η^2^ = 0.147, MSE = 0.884). Differences were found between the upright and the other orientations in the mirror version (all p < 0.05) and all combinations in the normal version (all p < 0.05) except for 0°-60° (p > 0.05). The orientation effect could be described by a linear and quadratic trend in both the mirror and normal versions. A summary of the orientation effects is presented in Table 2. A centro-parietal scalp distribution was found in the topographic mappings of ERP_RT_ for both versions (see Fig 6), and the orientation effect was more obvious for the normal version.

**Table 1:** Summary of significant main effects of version, orientation or laterality, and significant interactions of interest.

Component	Effect	Details
ERP_onset_	Version	F(1,21) = 5.384, p = 0.030, η^2^ = 0.006, MSE = 1.453
	Orientation	F(3,63) = 10.274, p < 0.001, ε = 0.830, η^2^ = 0.039, MSE = 1.645
	Laterality	F(1,21) = 4.669, p = 0.042, η^2^ = 0.023, MSE = 6.353
ERP_RT_	Version	F(1,21) = 17.696, p<0.001, η^2^ = 0.017, MSE = 0.777
	Orientation	F(3,63) = 24.640, p < 0.001, ε = 0.680, η^2^ = 0.104, MSE = 1.244
	Version×Orientation	F(3,63) = 8.422, p < 0.001, ε = 0.763, η^2^ = 0.020, MSE = 0.644

**Table 2:** Summary of orientation effects. Percentage of the variance explained by significant linear and quadratic trend components for each version.

Component	Version	Trend components	
		Linear	Quadratic
ERP_onset_	Mirror	72.6%, F(1,63) = 6.381, p = 0.014	n.s.
	Normal	86.1%, F(1,63) = 24.143, p < 0.001	n.s.
ERP_RT_	Mirror	82.0%, F(1,63) = 27.133, p < 0.001	16.5%, F(1,63) = 5.459, p = 0.023
	Normal	92.4%, F(1,63) = 78.517, p < 0.001	7.4%, F(1,63) = 6.355, p = 0.014

**Fig 7:**
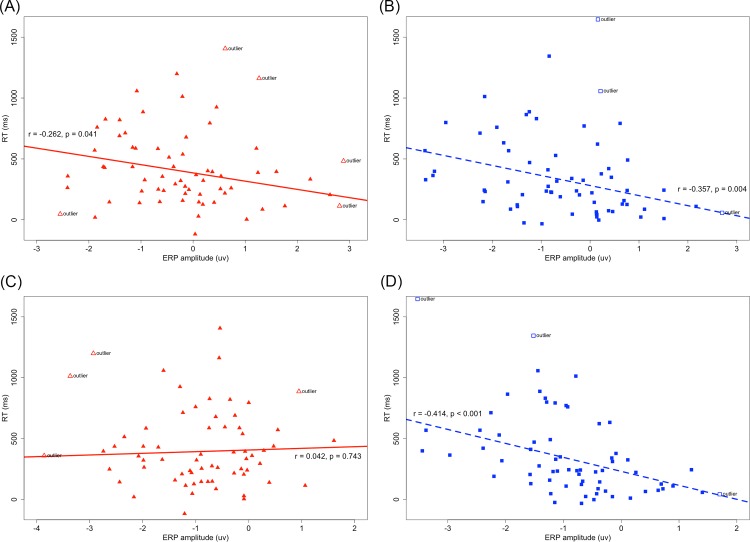
Association of individual response times to the rotated stimuli (60°, 120°, and 180°; N = 66) and the mean amplitude of (A) ERP_onset_ in the mirror version, (B) ERP_onset_ in the normal version, (C) ERP_RT_ in the mirror version, (D) ERP_RT_ in the normal version.

Previous studies have shown that individual performance in the mirror-normal letter discrimination task strongly correlates with the rotation-related negativity that is time-locked to the response (ERP_RT_) but not to the stimulus onset (ERP_onset_), although they all vary as a function of the orientation [2]. However, based upon our results, the mean ERP amplitude of 400–600 ms from the stimulus onset and 600–200 ms before the response were significantly correlated with individual response times to the rotated stimuli (60°, 120°, and 180°; N = 66) in both the normal (r = -0.316, p = 0.012 for ERP_onset_; r = -0.446, p < 0.001 for ERP_RT_) and mirror (r = -0.315, p = 0.013 for ERP_onset_; r = -0.349, p = 0.005 for ERP_RT_) versions. In order to control the non-specific effects of inter-individual differences in psychomotor speed and mean ERP amplitude [2], we then recalculated the correlation coefficient after both the mean ERP amplitude and individual response time were referenced to the baseline upright condition (by computing 60°-0°, 120°-0° and 180°-0° differences). Finally, the mean amplitude of ERP_onset_ remained correlated with individual performance in both the normal (r = -0.261, p = 0.034 before outlier detection; r = -0.357, p = 0.004 after outlier detection) and mirror (r = -0.107, p = 0.392 before outlier detection; r = -0.262, p = 0.041 after outlier detection) versions (see Fig 7(A) and 7(B)), while the significant correlation for ERP_RT_ was found only in the normal version (r = -0.498, p < 0.001 before outlier detection; r = -0.414, p < 0.001 after outlier detection) (see Fig 7(C) and 7(D)). Therefore, the amplitude modulation in both ERP_onset_ and ERP_RT_ mainly results from the mental rotation process rather than the increased duration/latency jitter among single trials [2]. In addition, the correlation between the mean ERP_RT_ amplitude and individual performance was more stable in the normal version, which could be explained by the influence of flipping in the mirror version.

**Fig 8:**
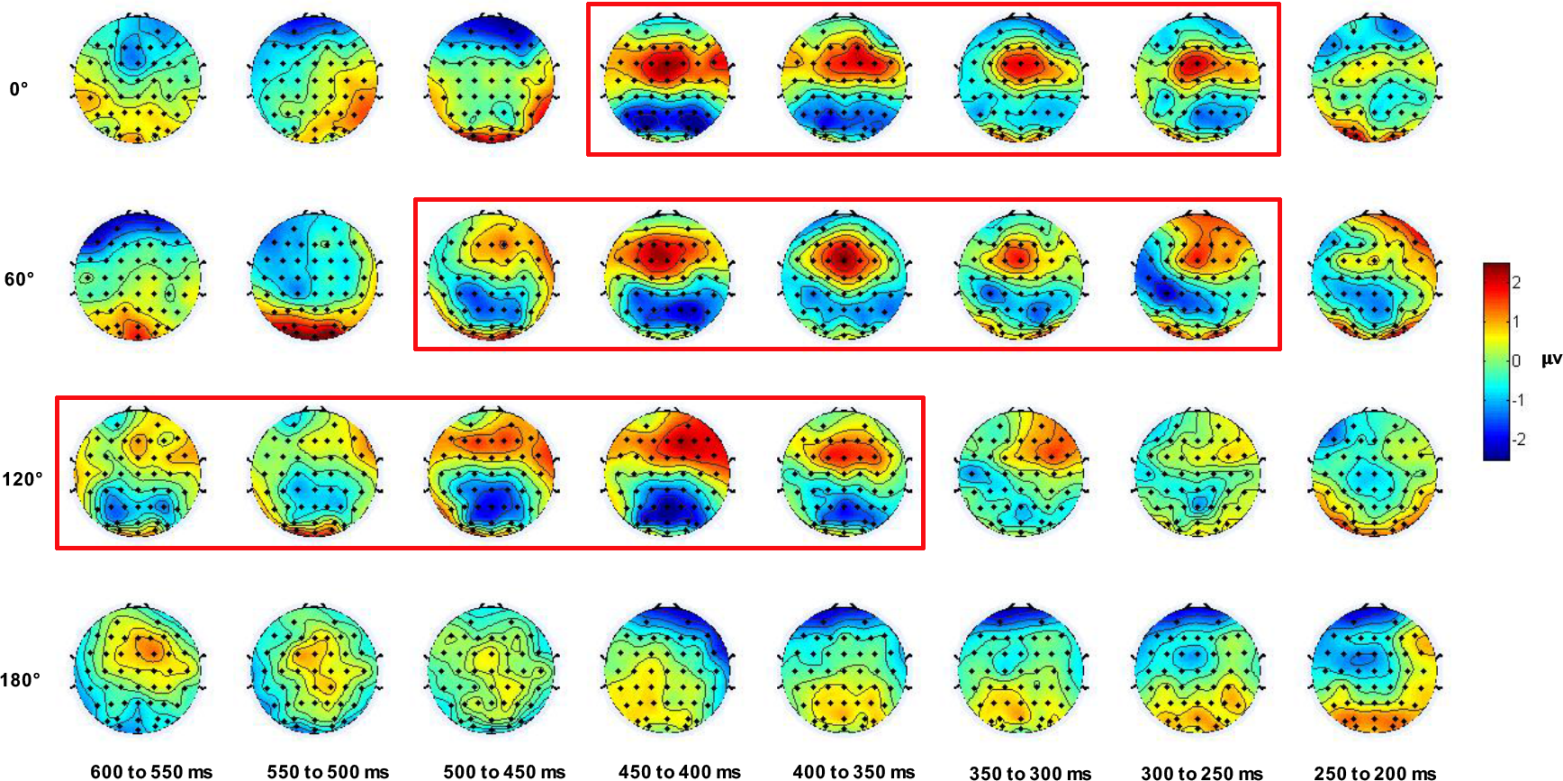
Topographic mapping of the differences in the mean mirror-normal amplitude at 0°, 60°, 120°, and 180° in 50-ms time windows from 600 to 200 ms before the response. Time windows with significant mirror-normal differences in the parietal region are marked by a red frame.

Fig 8 shows the topographic mapping of the mirror-normal difference for each orientation in 50-ms time windows from 600 to 200 ms before the response. A summary of the significant mirror-normal differences is presented in Table 3. First, for all time windows with significant differences, the mean ERP amplitude was smaller in the mirror than in the normal version (p < 0.05), excluding for the inverted condition at 400–350 ms, suggesting that the mental rotation process for the inverted orientation is likely different from that of other orientations. Additionally, there was no significant mirror-normal difference at any other time window for the inverted orientation, and therefore we temporarily set aside this condition. In a retrospective analysis of the late phase from the response time, we found that the mirror-normal difference first occurred at the orientation of 0° and 60° (300–250 ms), followed by 120° (400–350 ms). Similarly, this effect disappeared first in the upright orientation (450–400 ms), followed by the orientation of 60° (500–450 ms) and then 120° (600–550 ms). The overall duration of the significant mirror-normal differences was approximately the same across orientations: 200 ms for the upright orientation and 250 ms for orientations of 60° and 120°. Thus, we concluded that the late phase of mental rotation showed a delay in the mirror-normal difference over the scalp. No effects of laterality were observed for any time windows in the parietal region.

**Table 3:** Summary of significant mirror-normal differences for each orientation from 600 to 200 ms before the response.

Orientation	Time interval	Details
0°	450–400 ms	F(1,21) = 14.460, p = 0.001, η^2^ = 0.045, MSE = 1.999
	400–350 ms	F(1,21) = 11.950, p = 0.002, η^2^ = 0.056, MSE = 2.075
	350–300 ms	F(1,21) = 8.345, p = 0.009, η^2^ = 0.022, MSE = 1.215
	300–250 ms	F(1,21) = 6.395, p = 0.020, η^2^ = 0.020, MSE = 1.265
60°	500–450 ms	F(1,21) = 8.609, p = 0.008, η^2^ = 0.067, MSE = 3.246
	450–400 ms	F(1,21) = 20.370, p < 0.001, η^2^ = 0.093, MSE = 2.309
	400–350 ms	F(1,21) = 6.020, p = 0.023, η^2^ = 0.040, MSE = 2.937
	350–300 ms	F(1,21) = 5.913, p = 0.024, η^2^ = 0.052, MSE = 3.621
	300–250 ms	F(1,21) = 6.525, p = 0.019, η^2^ = 0.060, MSE = 3.895
120°	600–550 ms	F(1,21) = 13.780, p = 0.001, η^2^ = 0.063, MSE = 1.251
	550–500 ms	F(1,21) = 11.508, p = 0.046, η^2^ = 0.035, MSE = 2.899
	500–450 ms	F(1,21) = 5.741, p = 0.026, η^2^ = 0.085, MSE = 5.993
	450–400 ms	F(1,21) = 12.070, p = 0.002, η^2^ = 0.109, MSE = 3.171
	400–350 ms	F(1,21) = 4.831, p = 0.039, η^2^ = 0.047, MSE = 3.171
180°	400–350 ms	F(1,21) = 4.773, p = 0.040, η^2^ = 0.036, MSE = 1.517

## Discussion

In this study, we first comprehensively compared the early and late phases of the mirror-normal letter discrimination task in three aspects, including the orientation effect, the mirror-normal difference and correlations with individual performance. Then, another detailed analysis was implemented for every 50-ms time window to further assess whether there is a delay in the mirror-normal differences in the late phase of mental rotation. We attempted to confirm the hypothesis that flipping occurs in the late phase of mental rotation and explain the relationship between in-plane and out-of-plane rotation.

We replicated the widely accepted orientation effect that response time increases and mean ERP amplitudes become more negative with an angular deviation from the upright orientation, suggesting that mental rotation occurs in both the early and late phases of mental rotation [2, 16]. However, there were three major differences between the characteristics of ERP_onset_ and ERP_RT_. At first, the orientation effect of ERP_onset_ showed a better linear fit than ERP_RT_ in both the normal and mirror versions, suggesting that the early phase of mental rotation is mainly composed of in-plane rotation and that the late phase is influenced by processes other than in-plane rotation. This influence probably originates from the out-of-plane rotation or decision-making process. Second, the absence of a significant interaction of orientation and version in the early phase indicated that the additional process in the late phase has an effect on both orientation and version. Moreover, this effect tends to be more evident in the mirror version because the orientation effect for the rotation-related negativity in the mirror version seemed less clear than the effect in the normal version [9, 26, 28]. Consequently, the mirror-normal difference between orientations became more obvious in the late phase. Finally, though the mean amplitude of both ERP_onset_ and ERP_RT_ were negatively correlated with individual performance, the correlation for ERP_RT_ remained stable in the normal version rather than the mirror version. All of the above phenomena and related inferences helped us to conclude that flipping has an important influence on the late phase of mental rotation. The interaction between the flip component and in-plane rotation differentiates the late phase of mental rotation from the early phase. First, out-of-plane rotation occurs in the late phase after in-plane rotation to fully normalize the mirror version [9, 11], and different durations of interaction between two rotation processes at various orientations contribute to the significant interaction of version and orientation in the late phase. Meanwhile, there may be a mutual influence between in-plane and out-of-plane rotation for the mirror version, which leads to the ambiguous features of the orientation effect and the instability of correlations between the mean ERP_RT_ amplitude and individual performance in mirror trials.

Using the method adopted for ERP_onset_ in 2009 [28], another detailed analysis of the mirror-normal difference for each orientation in every 50-ms window at parietal sites showed that in the late phase of the mirror-normal letter discrimination task, the mirror-normal difference first occurred at orientations of 0° and 60° and disappeared at 120°. In the upright orientation, the discovery of similar mirror-normal difference again confirmed the existence of out-of-plane rotation in the late phase of mental rotation [9]. As the orientation increased and the proportion of trials employing mental rotation became larger [13, 14, 23], a longer in-plane rotation process appeared as expected. Due to the increase in the interaction between in-plane and out-of-plane rotation, the duration of the overall significant mirror-normal difference increased by approximately 50 ms at the orientation of 60°. Concurrently, the mirror-normal difference gradually approached the theoretically maximum duration because out-of-plane rotation correlated with individual rotation rates rather than orientations. Therefore, the duration at 120° was approximately equal to that at 60°. Interestingly, the significant mirror-normal difference at the orientation of 120° appeared 100 ms later than at 0° and 60° with respect to response time, which probably resulted from the longer post-rotation process associated with larger orientations. There are two possible explanations for the longer post-rotation process: (1) if subjects really make decisions by comparing the vertical and horizontal polarity of the character, with a larger orientation and greater difficulty in generating responses, then the spatial conflict between the polarity of the character-centered and viewer-centered coordinate system not only extends the in-plane rotation process but also prolongs the decision-making process [13, 14]; and (2) if there really is a tendency for subjects to prepare a normal response, with a larger orientation, the conflict between the expectation and the reality becomes more serious and likely extends the process of preparing a motor response [31]. Taking the interaction with in-plane rotation into account, out-of-plane rotation is likely to overlap with both the 2D rotation and the decision-making processes [9, 13, 31, 43], providing an explanation for the finding that the most obvious mirror-normal difference over the scalp appeared in the middle of the late phase (500–350 ms). In the inverted orientation, no similar mirror-normal difference (the mean ERP amplitude was smaller in the mirror than in the normal version) was observed for any time window, suggesting that the mental rotation process is likely different from other orientations [26, 28, 44]. This result is consistent with the phenomenon found in other studies on ERP_onset_ and could results from individual differences in rotation rates. If the mirror-normal differences were examined through ERP_onset_, individual differences in rotation rates could lead to larger variation of individual response times of in-plane rotation with larger orientations. As a result, substantial temporal variation of the flip component obscures the mirror-normal difference in the inverted orientation. While in this study, the mirror-normal differences were examined through ERP_RT_ and the flip component should line up with in-plane rotation for each orientation in the late phase for each subject. Therefore, the abnormal phenomenon in the inverted orientation is less likely to be affected by individual differences in rotation rates and there are another two possible explanations for this result through ERP_RT_. First, as previously reported, the mental rotation in and out of the plane probably occurs in parallel at more extreme orientations, which obscures the mirror-normal difference [28]. If two rotation processes occur in sequence at any orientations, the differences between the mirror and normal version should be found in the inverted condition [28]. Moreover, the parallel rotating mechanism reduced the response time for the inverted mirror trials. As a result, the advantage of response time for the normal version over the mirror version decreased at the orientation of 180° and the quadratic trend of the orientation effect disappeared in the mirror version. Second, a much larger drop in accuracy in the mirror version compared with the normal version indicates that there could be a speed/accuracy trade-off in the inverted mirror trials. Given the difficulty of managing an out-of-plane rotation in the inverted orientation, participants might fail to flip and choose an incorrect answer more often than in other orientations. Therefore, the difference between in-plane and out-of-plane rotation in the inverted orientation did not approach significance. Overall, further research should examine this abnormal phenomenon in the inverted orientation with a relatively high level of accuracy.

In summary, we performed a comprehensive analysis of the mirror-normal difference in both the early and late phases of mental rotation and deduced that flipping is more likely to occur in the late phase of the mirror-normal letter discrimination task and interacts with both in-plane rotation and the decision-making process. Although many previous studies in the early phase have indicated that flipping should exist and occur in the late phase of mental rotation, a comprehensive analysis of the late phase has the advantage of providing a more direct comparison of the in-plane and out-of-plane rotation and avoids the problems caused by the fact that post-rotation processing begins at different time points for different orientations. In other words, the mirror-normal difference in ERP_RT_ will precisely reveal the differences between the in-plane and out-of-plane rotation because the flip component would line up with the in-plane rotation for each orientation in the late phase. Therefore, based on previous reports in the early phase of the mental rotation, our behavioral and electrophysiological findings have improved the theoretical proof of flipping from a unique perspective and further confirmed the occurrence of out-of-plane rotation in the late phase. Meanwhile, the maximum duration of the mirror-normal difference in the late phase is theoretically closer to the accurate duration of out-of-plane rotation. Moreover, given that feature extraction and object recognition processes have more influence on the early phase of mental rotation while the decision-making process is mainly involved in the late phase [2], we could also acquire a better understanding of the relationship between in-plane rotation, our-of-plane rotation and other processes by comparing the different phases of the mirror-normal letter discrimination task in terms of three aspects, including the orientation effect, the mirror-normal difference and correlations with individual performance. As a result, our study in the late phase of mental rotation not only contributes to a direct inspection of the mirror-normal differences, but also helps to dissect the overall process of mental rotation.

## Supporting information

S1 File Relevant data underlying the findings described in manuscript. (ZIP)
